# Power Without Wires: Advancing KHz, MHz and Microwave Rectennas for Wireless Power Transfer with a Focus on India-Based R&D

**DOI:** 10.3390/s26010317

**Published:** 2026-01-03

**Authors:** Shobit Agarwal, Ananth Bharadwaj, Manoj Kumar, Antonio Iodice, Daniele Riccio

**Affiliations:** 1 Department of Electrical Engineering and Information Technology, University of Naples “Federico II”, 80125 Napoli, Italy; shobitagarwal@ieee.org (S.A.); dariccio@unina.it (D.R.); 2 Department of Electrical and Electronics Engineering, Birla Institute of Technology and Science, Dubai 333031, United Arab Emirates; ananth@dubai.bits-pilani.ac.in; 3 Department of Electrical Engineering, Indian Institute of Technology, Kanpur 208016, India; manoj95692@gmail.com

**Keywords:** wireless power transfer (WPT), rectennas, metasurfaces, mm-wave, efficiency, beamforming, India R&D

## Abstract

Wireless power transfer (WPT) technologies are advancing rapidly, yet their development trajectories within specific regional contexts remain underexplored. This review synthesizes India’s contributions to both near-field and far-field WPT research. We conducted a systematic literature survey spanning 2018–2024 to identify dominant technological themes, benchmark performance against global standards, and analyze innovation patterns within India’s research ecosystem. The review reveals a consistent focus on robust, cost-effective, and context-appropriate designs across both domains. In near-field WPT, Indian research emphasizes misalignment-tolerant magnetic coupling and high-frequency power converters for applications including electric vehicle charging and biomedical implants. In far-field WPT, progress is evident in rectenna architectures that enhance angular coverage and efficiency, particularly for IoT networks. We consolidate quantitative performance metrics from the literature to establish reference benchmarks and delineate persistent research gaps. We propose a forward-looking research agenda aimed at aligning WPT innovation with India’s sustainable development goals and energy accessibility challenges. This analysis provides a foundation for understanding how regional ecosystems shape technological priorities and offers insights for global WPT development.

## 1. Introduction

The development of WPT has evolved along two distinct technological trajectories. Near-field (non-radiative) methods operate through inductive or capacitive coupling at kilohertz to megahertz frequencies, while far-field (radiative) approaches use propagating electromagnetic waves from hundreds of megahertz to several gigahertz [1,2,3,4,5]. These paradigms serve complementary application spaces, each with specific advantages and limitations. Near-field systems achieve high power transfer efficiency over short distances but are highly sensitive to spatial alignment between transmitter and receiver coils [6,7]. Conversely, far-field techniques enable operation over longer distances with flexible receiver placement, though with reduced end-to-end efficiency and dependence on receiver orientation [8,9]. The commercial progress of near-field WPT has been supported by standardization efforts from consortia including the Wireless Power Consortium (Qi) and the AirFuel Alliance [10,11]. Far-field WPT continues to advance through academic research and industrial prototypes that gradually extend performance boundaries.

Regional innovation ecosystems significantly influence WPT development. Local infrastructure constraints, market demands, and institutional capabilities shape research priorities and technological directions [12,13,14,15]. India has become a notable contributor to this global landscape, with academic institutions, national laboratories, and technology startups advancing both fundamental research and application-specific implementations across near- and far-field modalities [16,17,18]. A characteristic feature of Indian WPT research is its emphasis on robustness, affordability, and adaptation to diverse operational environments. These priorities address practical challenges including variable alignment precision in electric vehicle charging scenarios [2,19,20], the need for maintenance-free power solutions for distributed IoT sensor networks [8], and the stringent reliability requirements of biomedical devices in resource-limited settings [21,22].

Multiple reviews document global advances in WPT technologies [1,2,23,24,25,26,27,28,29,30,31,32,33,34,35,36,37], yet systematic analyses of how specific regional ecosystems, particularly those in emerging economies, contribute distinctive innovations or address localized challenges remain limited. This gap is important because the practical implementation of WPT systems depends substantially on contextual factors including grid stability, ambient environmental conditions, infrastructure availability, and socioeconomic constraints. Additionally, while near-field and far-field WPT are often studied as separate research domains, technical convergence is emerging through hybrid architectures that combine the spatial precision of near-field coupling with the extended operational range of far-field radiation.

This review addresses these gaps by presenting a comprehensive analysis of India’s contributions to both near-field and far-field WPT research from 2018 to 2024. We systematically examine the technological evolution within this ecosystem, identify dominant thematic priorities, and benchmark reported performance against international standards. Specifically, this review aims to achieve the following:

1. Synthesize near-field WPT advancements from India, with focus on misalignment-tolerant magnetic coupling systems, adaptive power electronics, and applications in electric vehicle charging and biomedical devices.
2. Analyze far-field WPT contributions, including rectenna designs with enhanced angular coverage, metasurface-assisted focusing elements, and implementations for IoT and sensor networks.
3. Examine emerging convergence between near-field and far-field approaches within the Indian research context, identifying hybrid architectures and adaptive interfaces.
4. Quantify performance metrics from the literature to establish benchmarks and reveal trends specific to the Indian research ecosystem.
5. Identify persistent research gaps and propose a research agenda aligned with India’s sustainable development objectives and technological self-reliance goals.

The paper’s structure is as follows: Section 2 details the systematic methodology employed. Section 3 reviews developments in high-frequency near-field WPT systems. Section 4 analyses advancements in microwave rectennas for far-field applications. Section 5 identifies research gaps and outlines future directions. Section 6 presents conclusions.

## 2. Methodology

This review employs a systematic literature review methodology to ensure comprehensive coverage and analytical rigor. The process follows established guidelines for conducting structured reviews in engineering domains, with clearly defined protocols for literature identification, selection, and analysis. The methodology comprises four sequential phases: planning, search, screening, and synthesis.

### 2.1. Scope and Research Questions

The scope of this review encompasses peer-reviewed journal articles and conference proceedings published between January 2018 and December 2024. This timeframe captures recent developments in WPT technology while maintaining a focused corpus for analysis. The geographical scope is limited to research contributions with at least one author affiliated with an Indian institution. The review addresses three primary research questions.

- **RQ1** What are the dominant technological themes and innovation patterns in Indian near-field and far-field WPT research?
- **RQ2** How do Indian contributions compare with global benchmarks in terms of performance metrics and application focus?
- **RQ3** What research gaps and opportunities define the future trajectory of WPT development within the Indian ecosystem?

### 2.2. Search Strategy and Source Databases

A systematic search was conducted across three major electronic databases in IEEE Xplore, ScienceDirect, and SpringerLink. These databases were selected for their comprehensive coverage of engineering and applied physics literature relevant to WPT research. The search employed Boolean operators and keyword combinations to maximize recall while maintaining precision. The primary search string included terms such as *WPT*, *wireless energy transfer*, *WPT*, *rectenna*, *inductive charging*, *microwave power transmission*, and India-related keywords including *India*, *Indian*, *IIT*, *IISc*, *Indian Institute of Technology*, *Indian Institute of Science*, *BITS*, *NIT*, *National Institute of Technology*, *DRDO*, and *ISRO*. Additional searches were performed using specific technology-related terms such as *metasurface rectenna*, *misalignment tolerance*, *adaptive matching*, and *angular coverage* combined with India-related keywords to capture niche contributions.

### 2.3. Screening and Selection Criteria

The initial database search yielded 150 records. After duplicate removal, 125 unique records underwent title and abstract screening against predefined inclusion and exclusion criteria. The inclusion criteria required that articles present original research on WPT technology, either near-field or far-field, include at least one author with an Indian institutional affiliation, report quantitative performance metrics such as efficiency, power levels, or operating frequency, be published in peer-reviewed venues between 2018 and 2024, and be written in English. Articles were excluded if they were editorials, patents, or technical reports, focused exclusively on wireless communications without power transfer, contained minimal or unclear Indian research contribution, presented only simulation results without experimental validation, or were duplicate publications of the same research. Full-text assessment of 125 potentially relevant articles resulted in a final corpus of 89 studies for analysis. The selection process followed the PRISMA guidelines, with documentation of excluded studies and reasons for exclusion.

### 2.4. Data Extraction and Synthesis Framework

For each included study, data were extracted using a standardized template that captured publication metadata such as year, venue, and author affiliations; the WPT domain, distinguishing between near-field and far-field systems and specifying the technology focus; design parameters including operating frequency, coil or antenna geometry, and materials; performance metrics such as power transfer efficiency, output power, and misalignment tolerance; application context and key innovations; and comparative benchmarks against prior work. Quantitative data were then tabulated for statistical analysis, including calculation of efficiency ranges, frequency distributions, and performance trends, while qualitative data were thematically coded to identify recurring research themes, innovation patterns, and application priorities. The synthesis employed both descriptive analysis, summarizing the state of the field, and critical analysis, evaluating strengths, limitations, and gaps, to provide comprehensive insights into India’s WPT research landscape. This methodological rigor ensures that the survey offers a reproducible, evidence-based assessment of India’s contributions to WPT technology while maintaining transparency in the literature selection and evaluation processes.

## 3. Developments in High-Frequency Near-Field Charging Systems (KHz–MHz Range)

Analysis of 89 studies reveals 40 focus on near-field WPT, representing ∼45% of the corpus. Indian research concentrates in three application areas. Electric vehicle (EV) charging, consumer electronics, and biomedical implants represent the primary focus areas. The maturation of near-field WPT has been propelled by its adoption in EVs, consumer electronics, and biomedical implants [1,2,3]. This transition has been underpinned by research focused on efficiency, misalignment, and interoperability. Progress in high-frequency near-field systems has been driven by convergent advancements across three interconnected domains. Refined circuit-level modeling, innovative power electronics, and electromagnetic field manipulation represent these key areas. This section reviews seminal approaches within these domains, focusing on inductive coupling, which has dominated recent research and development.

### 3.1. Circuit-Level Modeling and Mutual Inductance Estimation

Accurate characterization of the magnetic coupling link, quantified by mutual inductance, represents the foundational step in WPT system design. The primary challenge lies in the dynamic nature of mutual inductance, which varies with spatial misalignment and the presence of nearby objects. Early research focused on deriving analytical expressions for mutual inductance, even for complex layered or irregularly shaped structures [38]. For practical systems, the research community has increasingly turned to dynamic, real-time estimation techniques. A prominent strategy uses auxiliary sensing systems, such as auxiliary pickup coils and sensing loops, which provide data that enables the system to auto-tune during operation [39,40].

Furthermore, the use of scattering parameters (S-parameters) has gained traction as a practical method for experimental validation and for estimating coupling strength to fine-tune impedance matching in real time [22,41]. The impact of the environment is also a critical factor, with recent studies mapping how shielding materials, nearby metal objects, and cross-talk influence performance in embedded setups [42,43]. A significant trend in integration involves the use of self-resonant conductive traces printed directly onto Printed Circuit Boards (PCBs). These operate in the Industrial, Scientific, and Medical (ISM) radio bands to reduce component count while improving quality factor and frequency stability [44].

### 3.2. Advancements in Power Electronic Converter Systems

Power electronic converters that drive transmitter coils have evolved from simple oscillator circuits to controlled systems designed for efficiency and robustness. The core challenge involves generating high-frequency, high-purity AC current while maintaining high efficiency across a wide range of load and coupling conditions. Research has delivered single-stage inverters and resonant topologies tailored specifically for wireless charging [33]. For applications like EVs, where bidirectional power flow is essential, new converter architectures handle both charge and discharge with minimal losses [45,46,47].

Scalability represents another theme. Modular converter systems that can drive multiple coils simultaneously are being developed, enabling larger charging surfaces or powering arrays of receivers [48,49]. The design of compensation networks is critical. Inductor-Capacitor-Capacitor (LCC) or Capacitor-Inductor-Capacitor (CLC) arrangements can reduce reactive power circulation, providing efficiency improvement [50]. The integration of adaptive switching logic directly into the magnetic field control loop represents an approach to maintaining system stability under shifting loads or receiver misalignment [32].

### 3.3. Magnetic Field Forming and Misalignment Compensation Techniques

**Figure 1:**
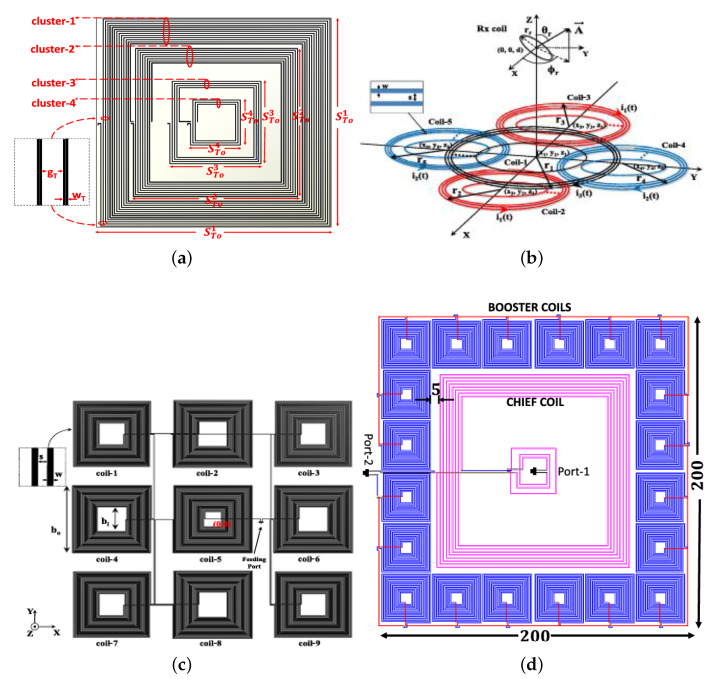
Recent near-field coil structures developed by Indian researchers [7,53,54,56] in order of display.

**Figure 2:**
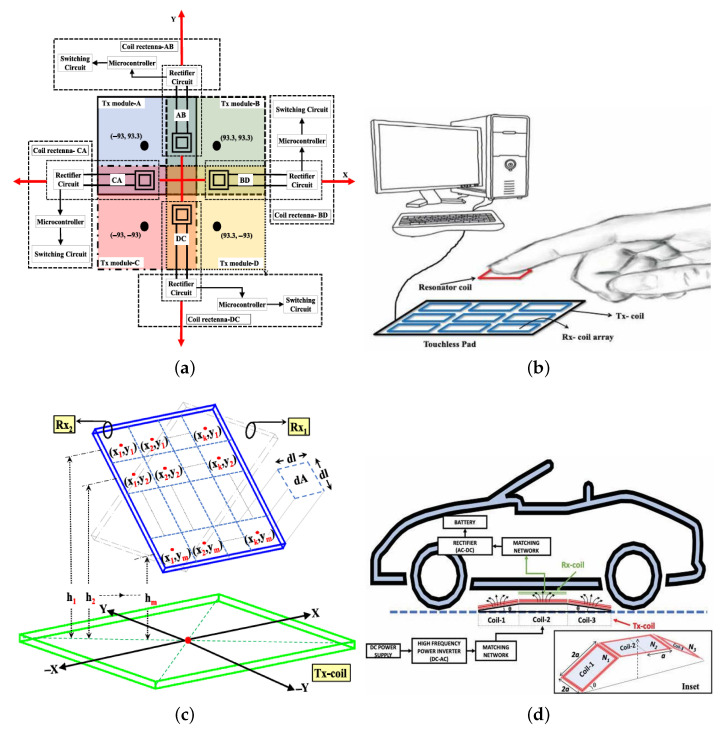
Recent near-field coil structures developed by Indian researchers [6,55,57,58] in order of display.

The most active area of innovation involves overcoming spatial misalignment. The research community’s response has shifted from passive tolerance through coil design to active field manipulation, termed “magnetic field forming.” Passive approaches have explored innovative coil geometries, including transmitter arrays that generate orthogonal field components to create a wider “magnetic sweet spot” [51]. The use of relay coils to bridge gaps caused by lateral misalignment represents another technique, with clustered relays maintaining solid coupling even with significant movement or poor alignment [7,52]. Other designs include distributed coil networks, dynamic relay switching, and smart pads with embedded sensors that track receiver position and adjust accordingly [53,54,55] as shown in Figure 1 and Figure 2.

The most transformative advances involve active field shaping. For applications where receivers may spin or tumble, generating elliptically polarized fields ensures consistent power delivery regardless of rotation [56]. This concept extends to systems that synthesize full three-dimensional field patterns using multiple transmitters, making orientation largely irrelevant [59]. The most sophisticated systems integrate localization data to sense receiver pose and dynamically reconfigure field shape and direction, creating a closed-loop “aim-and-power” system essential for applications like surgical implants or drone landing pads [60,61,62].

### 3.4. Synthesis

**Figure 3:**
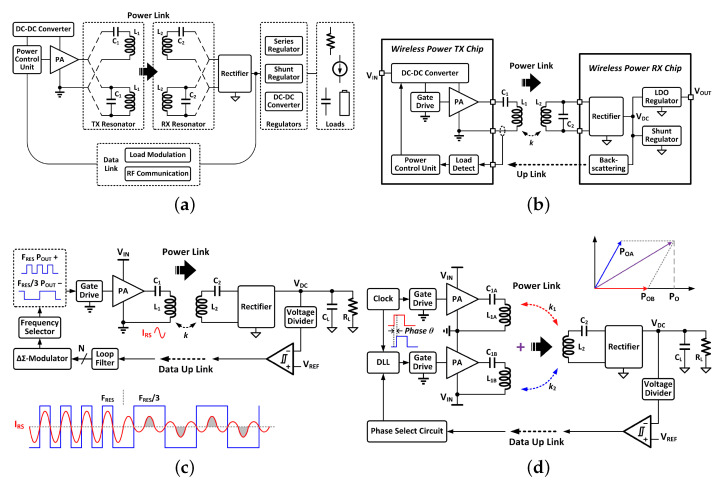
(**a**) The block diagram of a non-specific inductive WPT system [63] (**b**) A typical inductive WPT system with linear regulators and data up-link through load modulation for power control [63] (**c**) A WPT system with output power controlled by transmission frequency hopping [64] (**d**) A WPT system with output controlled by the phase difference of two transmitters [65].

The trajectory of research in high-frequency near-field WPT demonstrates evolution from isolated component optimization to a system-level approach. The integration of dynamic sensing, adaptive power electronics, and programmable field-forming antennas represents the current state. Challenges persist, including management of electromagnetic interference (EMI) at higher frequencies, ensuring biocompatibility for implantable devices, and developing cost-effective solutions for large-scale deployment. The convergence of these techniques smart modeling, efficient conversion, and intelligent field control provides a framework for addressing these remaining hurdles (see Table 1 and Figure 3).

While near-field WPT excels in high-efficiency, short-range power delivery, far-field WPT addresses complementary needs low-power, longer-range, and orientation-flexible energy transfer for distributed IoT sensors and remote monitoring. The next section transitions to radiative (far-field) approaches, focusing on rectenna design innovations.

## 4. Microwave Rectennas

### 4.1. Overview and Application Focus

From the analyzed corpus, 40 studies (45%) focus on far-field WPT systems. Unlike the near-field domain, where research concentrates on EV charging, far-field research primarily targets low-power applications. Internet of Things (IoT) sensor networks, radio-frequency identification (RFID) systems, and biomedical devices represent the main application areas. This distribution reflects the current technological maturity and practical constraints of radiative power transfer for energy-constrained applications (see Table 2).

### 4.2. Novel Rectenna Designs

**Figure 4:**
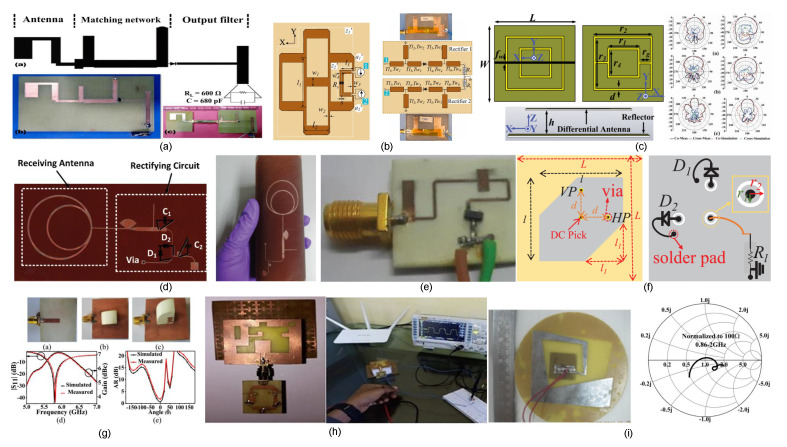
Representative rectenna topologies for far-field WPT: (**a**) dual-band sickle-shaped rectenna [4]; (**b**) dual-port grid array rectenna with harmonic reradiation capability [15]; (**c**) triple-band differentially fed rectenna [8]; (**d**) flexible dual-band rectenna array for wide azimuth coverage [5]; (**e**,**f**) coupler-free integrated dual circularly polarized rectenna [66]; (**g**) circularly polarized dielectric resonator rectenna [67]; (**h**) broadband circularly polarized rectenna with artificial magnetic conductor backing [13]; (**i**) broadband bent triangular omnidirectional rectenna [9].

Recent advances in rectenna design from India have addressed key challenges related to efficiency, compactness, polarization sensitivity, and environmental adaptability. A highly efficient dual-band rectenna operating at 2.4 GHz and 5.8 GHz for RF energy harvesting is reported in [4]. This design utilizes a novel sickle-shaped antenna, as shown in Figure 4a, achieving RF-DC conversion efficiencies of 63% and 54.8%. These deliver up to 3 V and 2.6 V, respectively, for low-power applications such as RFID tags and wearable devices. Similarly, a compact two-port grid array rectenna (GAR) with integrated 180° hybrid coupler functionality, shown in Figure 4b, is presented for WPT at 2.4 GHz [15]. The GAR achieves up to 72% RF-DC conversion efficiency and simultaneously radiates the third harmonic as a feedback signal, enabling real-time receiver monitoring in dynamic scenarios.

A triple-band differentially fed rectenna for RF energy harvesting in UMTS (2.1 GHz), WLAN/Wi-Fi (2.4–2.48 GHz), and WiMAX (3.3–3.8 GHz) bands is shown in Figure 4c and presented in [8]. The design combines a high-gain slot antenna with a Villard voltage doubler-based rectifier, achieving up to 68% RF-DC efficiency using a three-tone signal and measured efficiencies of 53%, 31%, and 15.56% across the three bands. A cylindrical-shaped dual-band flexible rectenna array, shown in Figure 4d, is proposed for RF energy harvesting in LTE (1.8 GHz) and Wi-Fi (2.45 GHz) bands [5]. The design employs four dual-band subsystems backed by an artificial magnetic conductor (AMC), achieving 40% RF-DC conversion efficiency at an input power of 12 dBm while maintaining compactness and flexibility for wide azimuth coverage.

A fully integrated dual circularly polarized rectenna (DCPR), shown in Figure 4f, is proposed for polarization-insensitive WPT to IoT nodes [66]. The design eliminates hybrid couplers and matching networks, achieving 35.13% PCE at −10.07 dBm. This represents a 126% improvement over conventional HC-based rectennas while ensuring miniaturization. A compact circularly polarized DR-rectenna, shown in Figure 4g, is proposed for RF energy harvesting at 5.8 GHz [67]. The design employs a 90° twisted quarter-sectored CDRA and a single shunt diode rectifier, achieving a peak RF-DC conversion efficiency of 70.5% at an input power of 5.75 dBm. A broadband rectenna, shown in Figure 4h, integrates a CPW-fed circularly polarized antenna with an AMC and a WRCN-based rectifier for battery-less indoor IoT sensors [13]. The design achieves 65% RF-DC conversion efficiency at an input power of 7 dBm and enables capacitor charging within 2 ms in a Wi-Fi environment. A broadband bent triangular omnidirectional rectenna, shown in Figure 4i, is proposed for RF energy harvesting across 850 MHz–1.94 GHz [9]. The design supports dual polarization and achieves peak efficiencies of 60% at 980 MHz and 17% at 1800 MHz, delivering up to 3.76 V at 25 m using a two-element array.

Compact multiport rectennas (N-LPR) enable polarization-insensitive WPT for IoT nodes [68]. Further innovations include 3D-printed biodegradable rectennas (67% efficiency) [69] and bend-tolerant arrays (40% efficiency) [5]. Dual circularly polarized rectennas (DCPR) and octa-port systems demonstrate enhanced power conversion efficiency (PCE) at ultra-low incident power levels [70]. A multifunctional integrated simultaneous information and power receive achieves 76% RF-DC efficiency [14]. Furthermore, a 90° twisted dielectric resonator exhibits 72.5% PCE at 5.8 GHz [67], biodegradable dual-band harvesters demonstrate 72.2%/51.21% efficiency at 2.4/5.2 GHz [12], and dual-band rectifiers achieve 54.9%/42.3% efficiency at 3.5/5.8 GHz [18]. μWsense rectenna operates at 5.2 GHz and has been tested up to 2 m [17]. RFEH systems provide ∼2 mW of output [16], and wide-range rectifiers demonstrate 79% PCE at 4 dBm with a 38 dB dynamic range [71].

Overall, recent rectenna designs from India demonstrate progress in addressing efficiency, compactness, and polarization sensitivity. Comparative analysis reveals trade-offs between multi-band versatility and conversion efficiency, as well as between flexible form factors and gain enhancement. Designs employing AMC-backed CP antennas [13] achieve indoor adaptability, while DCPR architectures [66] excel at ultra-low power levels, making them suitable for IoT nodes. Multi-band rectennas such as [8] enable harvesting from diverse sources but suffer efficiency degradation at higher frequencies, indicating a trade-off between versatility and performance. Efficiency at higher frequencies and under dynamic conditions remains a challenge.

### 4.3. Angular Misalignment Mitigation

**Figure 5:**
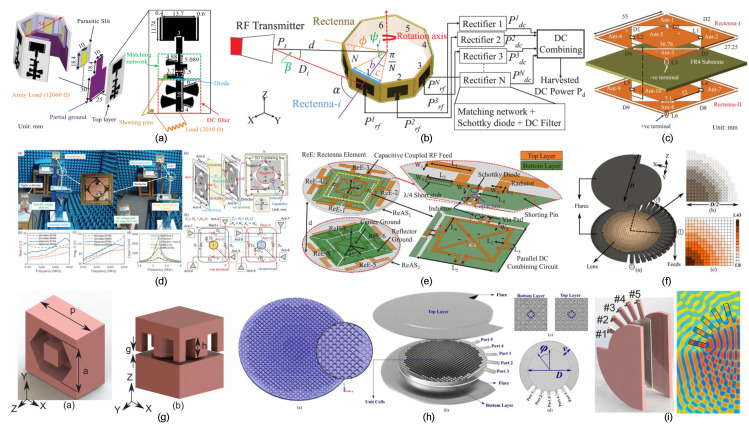
Comparative overview of advanced rectenna architectures for mitigating angular misalignment and enhancing energy capture: (**a**) a six-sector rectenna design [72]; (**b**) an eight-sector rectenna configuration [73]; (**c**) a compact isotropic rectenna [75]; (**d**) an isotropic rectenna operating without DC combining networks [76]; (**e**) a stacked rectenna element [77]; (**f**) a multi-beam rectenna system based on a Luneburg lens [78]; (**g**) an all-metal lens rectenna with microstrip feed [79]; (**h**) a directional multi-port Luneburg lens employing a dielectric metasurface [80]; and (**i**) a compact retroreflector with beam-switching capability [81].

Angular misalignment, defined as the deviation between the direction of incident RF signals and the rectenna orientation, causes power loss due to gain reduction. Omnidirectional rectennas [9] enable wide-angle harvesting however their low gain limits efficiency. To address this limitation, various multidirectional rectenna designs and analytical frameworks have been developed to enhance efficiency and mitigate angular misalignment. An analytical framework for designing angular misalignment-tolerant multisector rectenna arrays for wireless energy harvesting (WEH) is proposed, as shown in Figure 5a [72]. The framework derives constraints on the synthesized harvested DC power pattern to ensure uniform energy harvesting across the azimuth plane. Conventional patch-based rectennas require N=8 and N=12 sectors for single and 2×1 patch arrays, respectively, whereas a proposed multilayer wide-beam rectenna at 5.8 GHz achieves similar performance with only N=6 sectors, reducing size by 36.5%. Experimental results validate the framework, demonstrating RF-DC efficiencies up to 65.22% for single-source and 61.08% for multi-source scenarios, confirming its suitability for WSN deployment.

Similarly, a compact 3-D multisector orientation-insensitive WPT system, shown in Figure 5b, is proposed to mitigate PCE degradation caused by angular misalignment between the transmitter and rectenna along the azimuth plane [73]. The optimal number of sectors (N=8) is analytically derived using patch antenna design equations, and adjacent sector radiation patterns are configured to intersect at the ≈0.8-dB point for uniform coverage. Operating at 5.8 GHz, the proposed system achieves a 51.2% improvement in power harvesting capability compared to conventional patch antenna-based WPT systems, making it suitable for IoT and WSN applications. Further research includes 3D multisector arrays designed for uniform azimuth coverage in [74].

Various planar arrays have been reported to address hemispherical coverage issues in 3D space. A novel planar six-sector 3-D spherical coverage rectenna array for wireless energy harvesting is proposed [82]. The design employs two-layer PCB technology and integrates endfire and tilted bore-sight patch antenna elements within each sector to achieve uniform RF power harvesting in both azimuth and elevation planes. Six such sectors are radially combined using a low-loss parallel DC combining circuit, eliminating the need for matching networks and DC-LPFs, thereby reducing insertion losses and system size. Operating at 5.8 GHz, the endfire and bore-sight rectenna elements achieve RF-DC conversion efficiencies of 69.1% and 65.28%, respectively, delivering up to 280 mV from a single RF transmitter (EIRP ≈ 30 dBm). The proposed design provides orientation-insensitive energy harvesting suitable for IoT applications such as smart homes and wireless sensor networks. A fully integrated planar eight-sector rectenna array is proposed for microwave power transfer (MPT) to IoT sensor nodes [83]. The design achieves nearly uniform 3-D spherical DC coverage using radially arranged endfire elements and a multiarms bore-sight element, delivering up to 600 mV with RF-DC efficiencies of 48.47% and 47.32% at 5.8 GHz, making it suitable for orientation-insensitive wireless powering. Further research includes a hybrid polarization-insensitive rectenna array that achieves 58.9% efficiency with isotropic coverage [84]. Stacked 45°-offset 3D arrays offer 3D coverage [77], while DC-combining-free miniaturized rectennas using mirror-like structures deliver 2300 mV DC at 2.45 GHz [75]. A 5.2 GHz dual-layer design achieves 60.5% efficiency and 1600 mV output through integrated full-wave rectification [76].

The results across these multisector and isotropic rectenna architectures reveal a performance trend between angular coverage, rectification efficiency, and circuit complexity. Systems optimized for full 3-D spherical coverage generally achieve $\eta_{\mathrm{RF}-\mathrm{DC}}$ values in the 45–70% range but require multiple sectors and complex combining networks, whereas planar six-sector or hybrid two-layer layouts offer nearly comparable efficiencies (∼60–65%) with ∼30–40% lower footprint. A normalized comparison of $\eta_{\mathrm{RF}-\mathrm{DC}}$ per covered steradian suggests diminishing efficiency gains beyond eight sectors, implying that six to eight sectors represent an optimal balance between coverage and conversion performance. Furthermore, designs eliminating DC-combining networks (e.g., mirror-based rectennas) demonstrate that structural simplification can recover up to 10% efficiency otherwise lost to combining losses.

A comparative analysis of the multisector and isotropic rectenna architectures reveals a trade-off between angular coverage, rectification efficiency, and system complexity. Designs optimized for full 3D spherical coverage typically achieve RF-DC efficiencies of 45–70% but require multiple sectors and complex combining networks. In contrast, planar six-sector or hybrid two-layer layouts offer comparable efficiencies (∼60–65%) with a 30–40% reduction in footprint. The diminishing efficiency gains beyond eight sectors suggest that six to eight sectors represent an optimal balance between coverage and performance. Moreover, architectures that eliminate DC-combining networks demonstrate that structural simplification can recover up to 10% efficiency otherwise lost to combining losses.

### 4.4. Metasurface-Assisted Lens Based Rectenna

Metasurface-assisted RF energy harvesting systems offer improved PCE compared to traditional rectennas [78,79,80,85,86,87]. A rectifier-integrated Luneburg lens for K-band WPT applications is proposed, as shown in Figure 5f [78]. The lens employs glide-symmetric metasurface layers and five tapered waveguide launchers for multi-directional energy collection. Integrated rectifying circuits achieve a peak gain of 17.2 dB and a PCE of 63% at 24 GHz with an input power of 9.5 dBm, making it suitable for high-frequency wireless energy harvesting.

Metasurface rectennas offer broadband operation and dual-polarization capability for versatile energy harvesting [85], along with high-gain resonant cavities for low-power signal harvesting [86]. Integration of artificial magnetic conductors (AMCs) with dual-polarized arrays and metasurface-assisted lenses enhances RF-to-DC conversion efficiency while minimizing footprints. These systems enable dynamic energy capture from multiple directions through multi-beam Luneburg lenses using artificial dielectrics. This approach addresses the mm-wave paradox where available power density ($P_{\mathrm{mmW}}\propto f^{2}$) coexists with significant propagation losses ($\alpha_{\mathrm{mmW}}\propto f^{2}$). The inherent design flexibility makes metasurface rectennas ideal for power-constrained applications, including IoT sensors, WSNs, and RFID tags.

Recent advances focus on mm-wave implementations where higher power density offsets increased propagation losses. Multi-beam Luneburg lenses with artificial dielectrics [78,79,87] enable simultaneous energy capture from multiple directions, while 3D-printed low-loss variants [80] maintain compactness for 5G applications. Figure 5 illustrates such systems with DC combining mechanisms. However, lateral dimensions remain challenging; retro-directive compact lenses [81] address space constraints at the cost of reduced coverage range. While these designs have been validated at mm-wave frequencies, they require experimental verification to establish real-world performance, particularly regarding trade-offs between efficiency-bandwidth and size in practical deployments.

Across the reported metasurface-assisted rectenna architectures, a consistent trade-off emerges between gain enhancement, angular coverage, and fabrication complexity. Multi-beam Luneburg and Rotman lens systems achieve peak $\eta_{\mathrm{RF}-\mathrm{DC}}$ values of 60–76% at 24–28 GHz, offering 100°–130° angular coverage, whereas simpler 3D-printed or retro-directive lenses maintain efficiencies around 40–55% with substantially reduced footprint. When normalized to aperture area, metasurface-based rectennas provide a 1.5–2× gain improvement over planar multidirectional arrays, primarily due to the passive focusing capability of gradient-index (GRIN) or glide-symmetric media. However, these benefits diminish beyond a critical lens diameter of ∼$3\lambda_{0}$ due to spillover and polarization mismatch losses. Comparative analysis indicates that integrating artificial magnetic conductors (AMCs) or frequency-selective surfaces (FSSs) improves $\eta_{\mathrm{RF}-\mathrm{DC}}$ by 8–12% through impedance stabilization across wide incident angles. Consequently, the most practical metasurface-assisted solutions are those balancing moderate gain (≈17–20 dBi) and partial angular coverage (up to 120°) while preserving planar manufacturability. Such systems provide an optimal compromise between efficiency, compactness, and alignment tolerance for future 5G-powered IoT and wireless sensor networks.

### 4.5. Scalable Rectenna Systems for Adaptive WPT

Scalable rectennas address deployment challenges in IoT networks where heterogeneous nodes require power levels from microwatts to milliwatts while accommodating spatial constraints and polarization sensitivity [88,89]. These systems enable adaptive energy harvesting with high efficiency in compact form factors. Kumar et al. [88] present a modular 3D architecture operating at 5.8 GHz featuring integrated conjugate matching that eliminates external networks. Complementarily, Gupta et al. [89] implement sequentially rotated unit cells with harmonic-suppressing conjugate matching, achieving 54.3% PCE at −8 dBm with polarization insensitivity. Configurations demonstrate series-connected modules delivering 233.75 μW at 46.5% efficiency, and parallel modules achieving 47.5% efficiency, both maintaining 75° 3-dB DC beamwidth for angular tolerance. Battery-less operation validates practical scalability for surface-mounted IoT sensors.

Modular design approaches facilitate system adaptation to specific application requirements while maintaining performance consistency across deployment scales. The integration of adaptive impedance matching within modular units addresses frequency detuning caused by environmental variations, a common challenge in real-world deployments. Polarization diversity achieved through sequential rotation techniques enhances energy capture from arbitrarily polarized incident waves, increasing system reliability in uncontrolled environments. Thermal management in densely packed modular arrays represents an ongoing research challenge, particularly for outdoor applications in tropical climates where ambient temperatures can exceed 40 °C.

Standardization of interconnection interfaces between modular units would accelerate commercial adoption by enabling interoperability across different manufacturers. Current research focuses on developing plug-and-play interfaces that maintain impedance matching while facilitating mechanical assembly. Energy management algorithms that dynamically reconfigure modular connections based on incident power levels and load requirements represent another active research direction. These algorithms optimize power delivery to multiple loads simultaneously while preventing overcharging or under-voltage conditions in energy storage elements.

Field validation of scalable rectenna systems in real-world Indian environments remains limited. Most reported results derive from laboratory measurements under controlled conditions. Future work should include long-term performance evaluation in diverse deployment scenarios, including urban, rural, and industrial settings. Environmental factors such as temperature fluctuations, humidity variations, and particulate contamination can significantly affect RF performance and require systematic characterization. Accelerated life testing under simulated environmental stress conditions would provide valuable data for reliability assessment and design refinement.

Economic considerations play a crucial role in determining the practical viability of scalable rectenna systems. Cost analysis should encompass not only component expenses but also installation, maintenance, and operational costs over the system lifetime. Comparative economic assessment against alternative power solutions, including batteries and wired connections, would provide a comprehensive perspective on deployment feasibility. Manufacturing scalability from prototype to mass production requires attention to materials selection, fabrication processes, and quality control protocols. Design for manufacturability principles should guide development to ensure cost-effective production at scale while maintaining performance specifications.

## 5. Future Research Directions and Strategic Agenda

The evolution of WPT in the Indian context necessitates addressing several interconnected research challenges spanning electromagnetic theory, power electronics, and system integration. These directions represent critical bottlenecks whose solutions will enable next-generation applications. The following strategic agenda outlines priority areas organized by implementation timeframe and technological maturity.

### 5.1. Near-Term Priorities (1–3 Years)

Immediate research should address the most critical gaps identified in our analysis while building upon demonstrated strengths. For near-field systems, priority should focus on developing high-power charging infrastructure exceeding 50 kW for electric buses and commercial vehicles. This requires research in scalable magnetic couplers with active cooling, grid-supportive power electronics capable of bidirectional operation, and advanced electromagnetic interference (EMI) mitigation for dense urban deployment. For far-field applications, research should concentrate on sub-6 GHz systems operating in the 868 MHz and 915 MHz bands to address indoor penetration and rural connectivity challenges. This includes developing efficient rectennas with electrically small antennas, investigating propagation characteristics in Indian urban and rural environments, and establishing regulatory frameworks for these frequency bands. Concurrent research should advance hybrid energy harvesting systems that combine RF energy harvesting with solar, thermal, or kinetic sources to enable maintenance-free IoT nodes. Standardization represents another critical near-term priority. Indian researchers should actively contribute to international standardization bodies including IEEE, IEC, and SAE, particularly in areas where local experience provides unique insights, such as operation in high-temperature environments or cost-constrained implementations. Development of India-specific test standards for WPT system certification would accelerate commercialization while ensuring safety and interoperability.

### 5.2. Medium-Term Directions (3–5 Years)

Medium-term research should advance toward more intelligent and integrated systems. Artificial intelligence-enabled WPT networks should be developed, utilizing machine learning algorithms for predictive maintenance, dynamic resource allocation, and adaptive beamforming. These systems would employ distributed sensor networks to monitor grid conditions, user behavior, and environmental factors, optimizing power transfer parameters in real time. Research should also explore millimeter-wave WPT systems in the 24–60 GHz range for specialized applications requiring high directivity, such as drone charging stations, industrial automation, and space applications. This requires advancements in millimeter-wave rectifier design, efficient power combining at high frequencies, and thermal management for continuous operation. Indian capabilities in satellite technology and defense electronics provide a strong foundation for this research direction.

Biomedical WPT systems represent another medium-term priority, requiring interdisciplinary collaboration with medical researchers. Focus areas include developing fully implantable systems with adaptive power control based on physiological monitoring, investigating long-term biocompatibility of implant materials under Indian climatic conditions, and establishing safety protocols specific to India’s healthcare infrastructure. Material science innovations should target sustainable and high-performance materials for WPT applications. Research should develop domestically sourced ferrite materials with optimized properties for different frequency ranges, explore 2D materials such as graphene and MXenes for flexible and transparent antennas, and advance additive manufacturing techniques for complex electromagnetic structures.

### 5.3. Long-Term Vision (5+ Years)

Long-term research should pursue transformative approaches that could redefine WPT technology paradigms. Quantum-enhanced energy transfer mechanisms should be investigated, exploring potential efficiency breakthroughs through quantum coherence, entanglement-assisted transfer, or topological protection against environmental interference. While speculative, early exploration in this area could position India at the forefront of next-generation energy technologies. Cognitive WPT networks should be developed that autonomously manage energy distribution across heterogeneous devices and applications. These networks would employ federated learning to optimize performance across multiple installations while preserving privacy, adapt to changing regulatory environments, and self-heal from component failures or environmental disruptions.

Research should also investigate space-based WPT systems for national energy security applications. This includes studying orbital power beaming for disaster recovery operations, developing high-efficiency space-to-ground power transfer systems, and investigating the regulatory and safety implications of such technologies. India’s space capabilities provide a unique advantage in this potentially transformative area. Fundamental research in new physical mechanisms for wireless energy transfer, including non-radiative mid-range coupling and evanescent wave amplification, could open new technological pathways beyond current near-field and far-field paradigms.

### 5.4. Cross-Cutting Enablers and Infrastructure Development

Several cross-cutting initiatives are essential to support all research directions. First, establishing national WPT test and certification centers with capabilities spanning from microwatt to megawatt power levels, near-field to far-field regimes, and laboratory to field conditions. These centers should develop standardized testing protocols and provide independent verification for commercial products. Second, creating open-access datasets and simulation tools for the Indian research community, including propagation models for Indian cities, material property databases under tropical conditions, and validated simulation frameworks for various WPT technologies. This would reduce duplication of effort and accelerate research progress. Third, fostering industry-academia collaborative platforms that address specific sectoral needs such as automotive, healthcare, consumer electronics, defense, and agriculture. Each platform should develop technology roadmaps, organize challenge grants, and facilitate pilot deployments. Fourth, developing human capital through specialized educational programs in WPT technology, including master’s and doctoral programs, professional certification courses for engineers, and interdisciplinary training that combines power electronics, electromagnetics, control systems, and application domain knowledge.

### 5.5. Implementation Roadmap and Stakeholder Roles

Successful implementation of this research agenda requires coordinated action across multiple stakeholders. Academic institutions should focus on fundamental research, talent development, and early-stage technology validation. Research laboratories such as CSIR, DRDO, and ISRO should address mission-oriented challenges and high-technology readiness level (TRL) development. Industry partners should drive commercialisation, scale-up, and market adaptation. Government agencies should provide strategic direction, funding mechanisms, regulatory frameworks, and public infrastructure support. A phased funding approach should balance short-term applied research addressing immediate market needs with long-term exploratory research pursuing breakthrough innovations. Public–private partnership models should be encouraged, particularly for high-risk, high-reward projects with potential for significant societal impact.

### 5.6. Alignment with National Priorities

This research agenda aligns strategically with India’s national priorities, including Atmanirbhar Bharat by developing domestic WPT technology capabilities, the National Electric Mobility Mission Plan through advanced EV charging solutions, Digital India via powered IoT infrastructure, and Sustainable Development Goals through efficient energy distribution systems. The proposed directions also support India’s climate commitments by enabling renewable energy integration and reducing transmission losses. By systematically addressing the identified research gaps while leveraging India’s comparative advantages, this strategic agenda aims to position India as a global leader in context-appropriate WPT technologies, contributing both to domestic development objectives and to the global advancement of WPT science and engineering.

## 6. Conclusions

This systematic review synthesizes India’s contributions to WPT research from 2018 to 2024. Our analysis of 89 peer-reviewed studies reveals distinctive innovation patterns within India’s research ecosystem, characterized by a focus on robustness, cost-effectiveness, and adaptation to local operating conditions. In near-field WPT, Indian research demonstrates particular strength in misalignment-tolerant systems, with innovations in phased coil arrays, adaptive relay networks, and optimized magnetic coupler geometries. These contributions address practical challenges in electric vehicle charging and biomedical implants. Far-field research exhibits notable advances in angular coverage and material integration, including multi-sector rectenna arrays, metasurface-assisted lenses, and flexible or biodegradable substrates optimized for IoT and sensor networks. Comparative analysis indicates that Indian contributions combine competitive performance with contextual adaptation, emphasizing suitability for tropical climates and resilience to grid intermittency. However, several research gaps persist. These include limited high-power near-field systems, underexplored sub-6 GHz far-field frequencies, scarce system-level integration studies, and insufficient attention to sustainability metrics and technology maturation pathways.

The proposed research agenda addresses these gaps while leveraging India’s strengths in cost-constrained innovation and experience with challenging environments. Near-term priorities focus on high-power EV charging, sub-6 GHz far-field systems, and standardization. Medium-term directions include AI-enabled networks, millimeter-wave WPT, and biomedical applications. Long-term vision encompasses quantum-enhanced transfer and cognitive networks. Cross-cutting enablers such as test infrastructure, open-access datasets, and specialized education are essential for implementation. This analysis provides a foundation for understanding how regional ecosystems shape technological priorities. India’s documented contributions represent important steps toward realizing WPT’s potential for equitable energy access, supporting sustainable development, and empowering innovation across sectors. Future progress will depend on systematic translation of research insights into practical solutions that address India’s unique energy challenges while contributing to global WPT advancement.

## Figures and Tables

**Table 1:** Characteristics of designs presented in Figure 1 and Figure 2.

Ref	Size (Tx)	Size (Rx)	Resonant Freq	Material
[7]	200 mm	100 mm	488.6 KHz	FR-4
[56]	59 mm	20 mm	500 KHz	FR-4
[53]	NA	NA	488.6 KHz	FR-4
[54]	200 mm	NA	488.6 KHz	FR-4
[55]	241.8 mm	93.2 mm	300 KHz	Litz Wire/ PCB Copper Wire
[57]	62 mm	18 mm	13.56 MHz	FR-4
[58]	200 mm	100 mm	400 KHz	FR-4
[6]	100 mm	100 mm	488.6 KHz	PCB Material

**Table 2:** Comparative overview of rectenna designs from Indian research (2018–2024).

Ref	Frequency Band(s)	Peak RF-DC Efficiency	Output Power/Volatge	Polarization	Key Innovation/Application
[4]	2.4, 5.8 GHz	63%, 54.8%	3 V, 2.6 V	Linear	Dual-band sickle-shaped antenna for RFID/wearables
[5]	1.8, 2.45 GHz	40% @ 12 dBm	—	Dual-band flexible	Cylindrical array with AMC for wide azimuth coverage
[8]	2.1, 2.4, 3.5 GHz	68% (3-tone)	—	Differential	Triple-band slot antenna + Villard doubler
[9]	0.85–1.94 GHz	60% @ 980 MHz	3.76 V @ 25 m	Dual	Bent triangular omnidirectional rectenna
[13]	Wi-Fi band	65% @ 7 dBm	—	Circular	AMC-backed CP antenna for indoor IoT sensors
[15]	2.4 GHz	72%	—	Linear	Grid array rectenna with harmonic feedback
[66]	5.8 GHz	35.13% @ −10.07 dBm	—	Dual CP	Coupler-free integrated DCPR for IoT nodes
[67]	5.8 GHz	70.5% @ 5.75 dBm	—	Circular	90° twisted dielectric resonator rectenna

## Data Availability

No new data were created or analyzed in this study.
